# Efficacy of Community-Based Exercise Therapy Among African American Patients With Peripheral Artery Disease: A Randomized Clinical Trial

**DOI:** 10.1001/jamanetworkopen.2018.7959

**Published:** 2019-02-15

**Authors:** Tracie C. Collins, Liuqiang Lu, Jasjit S. Ahluwalia, Nicole L. Nollen, John Sirard, Robert Marcotte, Spencer Post, Rosey Zackula

**Affiliations:** 1 Department of Preventive Medicine and Public Health, School of Medicine, University of Kansas Medical Center, Wichita; 2 Department of Behavioral and Social Sciences, School of Public Health, Brown University, Providence, Rhode Island; 3 Department of Preventive Medicine and Public Health, University of Kansas Medical Center, Kansas City; 4 Department of Kinesiology, Commonwealth Honors College, University of Massachusetts, Amherst; 5 Office of Research, School of Medicine, University of Kansas Medical Center, Wichita

## Abstract

**Importance:**

African American individuals are 2 times more likely than non-Hispanic white individuals to have peripheral artery disease (PAD). Structured community-based exercise therapy improves walking distance among patients with PAD, but these patients require motivation to adhere to therapy.

**Objective:**

To assess whether motivational interviewing (MI) is more efficacious than Patient-Centered Assessment and Counseling for Exercise (PACE) or control to improve walking distance in African American patients with PAD.

**Design, Setting, and Participants:**

In this 3-group randomized clinical trial, 174 African American patients with PAD were studied from May 1, 2012, to November 30, 2016, at health care centers, churches, and health fairs in Wichita, Kansas; Kansas City, Kansas, and Kansas City, Missouri.

**Interventions:**

Patients were randomized in a 1:1:1 fashion to 1 of 3 groups (57 to MI, 57 to PACE, and 60 to control). The 2 counseling interventions were delivered biweekly for 3 months and monthly for 3 months followed by a 6-month maintenance phase with limited contact. Control participants received a mailing at 3 and 9 months.

**Main Outcomes and Measures:**

The primary outcome was 6-month change in 6-minute walking performance. Secondary outcomes included 12-month change in walking performance and 6- and 12-month changes in quality of life.

**Results:**

A total of 174 African American patients (mean [SD] age, 64.2 [11.2] years; 128 [74.0%] female) were studied. At 6 months, mean (SE) change in walking distance by group was as follows: MI, −3.42 (4.55) m; PACE, 2.74 (6.00) m; and control, −0.18 (4.40) m. At 12 months, mean (SE) change in walking distance by group was as follows: MI, −7.75 (5.50) m; PACE, 13.75 (6.13) m; and control, −1.08 (5.73) m. Comparing each of the intervention arms (MI and PACE) with the control arm, no statistically significant increases in walking distance at 6 months (MI: change, −2.10 m; 95% CI, −16.54 to 12.35 m; PACE: change, 2.31 m; 95% CI, −11.36 to 15.97 m) or 12 months (MI: change, −5.56 m; 95% CI, −21.18 to 10.06 m; PACE: change, 14.24 m; 95% CI, −1.85 to 30.34 m) were found. Compared with MI, PACE resulted in a statistically significant increase in walking distance at 12 months of 19.80 m (95% CI, 3.33-36.28 m).

**Conclusions and Relevance:**

In a cohort of African American patients with PAD, MI was not efficacious in improving walking distance at 6 or 12 months. The results of this study do not support the use of MI to improve walking performance in African American patients with PAD.

**Trial Registration:**

ClinicalTrials.gov Identifier: NCT01321086

## Key Points

**Question:**

Is motivational interviewing efficacious for improving walking distance among African American patients with peripheral artery disease?

**Findings:**

In this randomized clinical trial of 174 African American patients with peripheral artery disease, no increases in walking distance were found when comparing motivational interviewing with Patient-Centered Assessment and Counseling for Exercise or control.

**Meaning:**

Among African American patients with peripheral artery disease, motivational interviewing did not improve walking distance in this study.

## Teaser

This randomized clinical trial assesses whether provision of educational materials with motivational interviewing or with Patient-Centered Assessment and Counseling for Exercise improves walking performance in African American patients with peripheral artery disease.

## Introduction

African American individuals are more than 2 times as likely as non-Hispanic white individuals to have peripheral artery disease (PAD)—atherosclerosis of the abdominal aorta and arteries of the lower extremities.^1^ Furthermore, African American individuals with PAD experience greater walking impairment (defined as a reduction in walking distance, speed, and/or stair climbing) and more severe disease compared with non-Hispanic white individuals.^2^ These identified disparities are largely attributed to lower levels of physical activity in African American individuals.^2^ Reduced physical activity is associated with an increased risk of mobility loss and a higher risk of functional decline, with subsequent inability to perform activities of daily living. Structured community-based exercise therapy, walking therapy conducted outside a rehabilitation setting and with minimal supervision, is potentially an excellent therapy for PAD, but patients must be motivated to walk. Because of low levels of physical activity, African American patients with PAD may benefit from motivational strategies to promote community-based walking therapy.

Several randomized clinical trials^3,4,5^ have demonstrated the clinical efficacy of motivational interviewing (MI) for physical activity. However, little is known about its benefits to motivate walking therapy in patients with PAD, particularly among African American patients who are most at risk of functional decline and lower-extremity amputations.^6,7,8^ We therefore performed a randomized clinical trial to determine the most effective counseling strategy to improve home-based walking in African American patients with PAD. We hypothesized that MI would be more efficacious than Patient-Centered Assessment and Counseling for Exercise (PACE) or control to improve walking distance in African American patients with PAD.

## Methods

### Participants

In this 3-group, intention-to-treat randomized clinical trial, 174 African American patients with PAD were recruited from May 1, 2012, to November 30, 2016, from health care centers, churches, and health fairs in Wichita, Kansas; Kansas City, Kansas; and Kansas City, Missouri. The recruitment schedule was extended to 42 months vs the originally planned 30 months given the principal investigator’s (T.C.C.) relocation. Inclusion criteria were African American (by self-report), resting ankle-brachial index (ABI) of 0.99 or less to assess for PAD, English speaking, and telephone access. We included patients with borderline PAD (ABI, 0.90-0.99) because their walking is often impaired.^9^ We excluded persons with 1 or more of the following: currently walking for exercise at least 3 days per week (ie, a PACE score ranging from 5-8, with 1 indicating precontemplation; 2, contemplation; 3, preparation; 4, action; and 5-8, maintenance to termination), major amputation (foot or lower leg) or critical leg ischemia (tissue loss, gangrene, or ulcers), rest pain with an ABI less than 0.4 without prior evaluation by a vascular surgeon, leg revascularization within 3 months of enrollment or plans for revascularization during the study period, supplemental oxygen use, or myocardial infarction within the preceding 3 months. Our recruitment approaches are detailed elsewhere.^10^ After participants were deemed eligible and provided written informed consent, they were randomized to 1 of 3 conditions and evaluated with a variety of assessment questionnaires. The interventions included telephone counseling sessions by trained personnel. The Human Subjects Committee at University of Kansas School of Medicine, Wichita, approved the study protocol. The trial protocol can be found in Supplement 1. This study followed the Consolidated Standards of Reporting Trials (CONSORT) reporting guideline.

Our planned enrollment of 204 was inflated to balance attrition, which we planned to hold to less than 15%. With a sample size of 174, we had 80% power to detect a mean difference of 40 m in change in the 6-minute walking distance (6-minute walking distance at 6 months minus 6-minute walking distance at baseline), comparing the MI and control arms. The calculations were based on the difference of half a city block (40 m) and using a Bonferroni correction for the multiple comparisons (ie, α was taken as .025). An estimate for the SD was taken as approximately 88 m, which was based on the variability observed in a study^11^ that reported changes during 6 months in a similar population with PAD. The primary comparison was among the MI, PACE, and control arms at 6 months; a secondary comparison was among the MI, PACE, and control arms at 12 months as well as between PACE and control arms at 6 and 12 months.

Participants were randomized to 1 of 3 study groups in a 1:1:1 fashion: control, MI, or PACE. A computer program generated the sequence, and at randomization, participants opened their envelopes to determine their group assignment. The research assistant measuring outcome data was masked to group assignment.

### Interventions

All groups received standard educational print material on managing PAD, including the importance of walking. In addition, the control group received mailings at 3 and 9 months to support continued engagement. They included a prepaid postcard for updating contact information. Participants randomized to the MI or PACE arm received telephone counseling sessions every 2 weeks for 3 months, then monthly for 3 months.

The PACE and MI participants received 1 instructional session on walking, conducted immediately after randomization. Participants were instructed to walk until their leg discomfort reached moderate intensity (a level of 3 on the claudication leg pain scale of 0-4, with 0 indicating no pain; 1, onset of pain; 2, mild pain; 3, moderate pain; and 4, severe pain).^12^ Persons with asymptomatic disease were instructed to walk until their exertion reached 7 to 8 (very hard) on the Borg 10-point scale of perceived exertion (with 1 indicating very easy and 10 indicating maximum difficulty)^13,14^ and then stop and rest until it subsided. Initially, participants were asked to repeat this cycle for the duration that they were able to walk on the treadmill during baseline assessment, then add a least 5 minutes to each session each week, until they could walk for at least 50 minutes 3 times a week or 30 minutes 5 times a week, independent of the number of times they had to stop during each session and independent of intensity.

#### Motivational Interviewing 

Motivational interviewing is a directive, client-centered counseling approach to elicit behavior change by assisting clients in exploring and resolving ambivalence.^15^ In this study, MI focused on 3 phases: exploring and dealing with resistance to walking; guiding and helping participants identify reasons, needs, and desires to increase walking; and choosing a goal, setting an action plan, and arranging follow-up. A master-level counselor (S.P.) trained in MI provided the intervention. All sessions were audiotaped and reviewed at supervision sessions provided every other week by a counseling psychologist (N.L.N.) and member of the Motivational Interviewing Network of Trainers. Audiotapes were reviewed using the OnePass MI fidelity assessment and supervision tool, which has high reliability and concurrent validity relative to the Motivational Interviewing Treatment Integrity system developed for real-world settings.^16^

#### Patient-Centered Assessment and Counseling for Exercise 

The PACE program is based on the transtheoretical model, which assumes that people make behavioral changes progressively and that they have different counseling needs at each stage. PACE targets known, modifiable determinants of physical activity, such as self-efficacy, social support, and perceived barriers to action.^17^ Using the PACE protocol, our counselors identified a patient’s current stage of change and, with a counseling script, tailored the recommendation to a patient’s needs. All sessions were audiotaped and reviewed at supervision sessions provided every other week by the project manager.

### Measures

The ankle-brachial index (ABI) was used to define PAD. During this assessment, a participant rested for 5 minutes and a 5-MHz handheld Doppler device with an attached stethoscope was used to measure systolic blood pressures in both brachial arteries and in both ankles (ie, the dorsalis pedis and posterior tibial arteries). The resting ABI was calculated based on the ratio of the ankle and arm pressures. For each leg, the ankle pressure was the higher of the dorsalis pedis and posterior tibial artery systolic blood pressures.^18^ The arm pressure used was the higher of the right and left brachial systolic pressures. The leg with the lowest ABI was the determining cut point for defining disease.

The short physical performance battery^19^ was used to assess balance and basic physical mobility. The short physical performance battery is an indicator of disability and mobility. Participants were asked to do a series of timed functional tests, with results scored based on the time. The score is measured on a 1- to 12-point scale in which a lower value indicates several limitations and a higher value indicates minimal limitations with physical performance. The maximum possible score is 12; participants with a total score of 10 or lower were eligible.

Potential study participants completed an exercise treadmill test with 12-lead electrocardiographic monitoring and blood pressure measurement; this test requires a constant treadmill speed with modest increases in grade every few minutes. Speed was kept constant at 2.0 mph with a 2% increase in grade every 2 minutes.^20^ If findings were concerning, participants were ineligible.

We used the Lifestyle and Clinical Survey to obtain sociodemographic and comorbidity data.^21^ The PACE score^17^ was used to identify a participant’s stage of readiness for exercise: participants chose 1 of 8 graded statements that best described their current level of and interest in physical activity.

### Outcomes

We used the 6-minute walk test to assess the primary outcome of walking distance, comparing baseline with 6 months. This test is a widely accepted and objective measure of walking distance.^22,23,24^ In contrast to treadmill testing, it provides information on patients’ ability to walk in the community; thus, it is a useful measure of the functional outcomes of our behavioral intervention to promote community-based walking. For the test, patients walked as many laps as possible around 2 cones 100 ft apart in a marked hallway. Patients were permitted to stop walking during the test, but time recording continued during the rest period. We recorded time and distance to onset of leg discomfort and total distance walked, measured in feet and converted to meters.

Secondary outcomes included quality of life and intrinsic-extrinsic motivation. Health-related quality of life was measured by the previously validated Medical Outcomes Study 12-Item Short Form Health Survey.^25,26,27,28^ The PAD-specific quality of life was measured using the Vascular Quality of Life questionnaire^29^ to obtain scores for participants’ level of activity, symptoms, pain, emotions, and social support.

Intrinsic motivation is a key concept when determining MI’s efficacy, and it was assessed with the Treatment Self-Regulation Questionnaire.^30,31,32^ The 15-item measure yields 3 main subscales of reasons why a respondent might begin or maintain exercise: (1) intrinsic (autonomous) motivation, (2) extrinsic (controlled) motivation, and (3) amotivation.

### Statistical Analysis

The primary and secondary outcomes, 6-month and 12-month change in 6-minute walking distance, were analyzed across the 3 groups. Multiple imputation was applied to replace missing values for nonresponse items using an imputation regression model with control for intervention, sex, baseline age, PAD severity, walking distance, and lowest ABI. Both χ^2^ and 2-sided *t* tests were used to compare categorical and continuous characteristics of participants across the 3 groups at baseline, respectively. A repeated-measures analysis of variance was used to compare changes in outcomes among baseline, 6-month follow-up, and 12-month follow-up between the interventions and the control group with adjustment for unbalanced data. The same analysis procedures were conducted on the additional exploratory (subgroup) analyses (eg, participants who completed the study). The analyses were performed using SAS statistical software, version 9.4 (SAS Institute Inc). *P* < .05 was considered to be statistically significant.

## Results

A total of 174 African American patients (mean [SD] age, 64.2 [11.2] years; 128 [74.0%] female) were randomized to 1 of 3 study protocols (57 to MI, 57 to PACE, and 60 to control). At 6 months, 52 participants remained in the MI group compared with 47 in the PACE group and 55 in the control group (Figure). By randomizing 174 participants, we met 85% of our goal of 204. Baseline participant characteristics are given in Table 1 and are reported for the overall cohort and by the originally assigned treatment group. No differences in baseline characteristics were found among the 3 groups. Mean (SD) baseline walking distance for the entire cohort was 357.55 (71.53) m. Mean (SD) walking distance by group was as follows: MI, 366.72 (72.85) m; PACE, 350.15 (70.61) m; and control, 355.85 (71.37) m. On the basis of multiple imputation (Table 2), at 6 months mean (SE) change in walking distance by group was as follows: MI, −3.42 (4.55) m; PACE, 2.74 (6.00) m; and control, −0.18 (4.40) m. At 12 months, mean (SE) change in walking distance by group was as follows: MI, −7.75 (5.50) m; PACE, 13.75 (6.13) m; and control, −1.08 (5.73) m. Comparing both MI and PACE with control, no statistically significant increases in walking distance were found at 6 months (MI: change, −2.10 m; 95% CI, −16.54 to 12.35 m; PACE: change, 2.31 m; 95% CI, −11.36 to 15.97 m) or 12 months (MI: change, −5.56 m; 95% CI, −21.18 to 10.06 m; PACE: change, 14.24 m; 95% CI, −1.85 to 30.34 m). When comparing PACE and MI at 12 months, there was a significant increase in walking distance of 19.80 m (95% CI, 3.33-36.28 m) (eTable in Supplement 2). Mean (SE) within-group changes at 6 months were as follows: MI, −3.42 (4.55) m; PACE, 2.74 (6.00) m; and control, −0.18 (4.40) m. At 12 months, mean (SE) within-group changes were as follows: MI, −7.75 (5.50) m; PACE, 13.75 (6.13) m; and control, −1.08 (5.73). Compared with control, there was a significant increase in mean walking distance at 12 months among participants randomized to PACE of 14.24 m (95% CI, −1.85 to 30.34 m) (eTable in Supplement 2).

**Figure.**
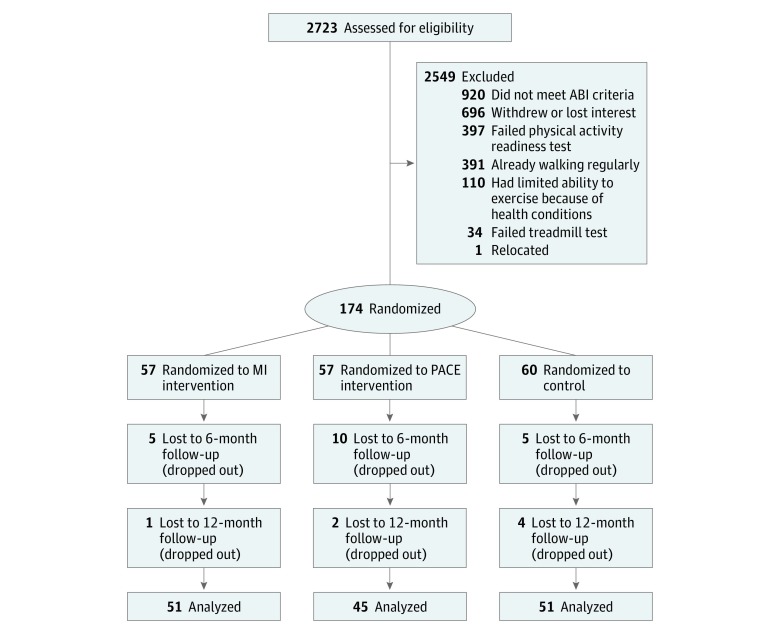
CONSORT Flow Diagram ABI indicates ankle-brachial index; MI, motivational interviewing; and PACE, Patient-Centered Assessment and Counseling for Exercise.

**Table 1.** Baseline Characteristics of Participants Randomized to MI or PACE vs Control^a^

Characteristic	Overall (N = 174)	MI (n = 57)	PACE (n = 57)	Control (n = 60)	*P* Value
Age, mean (SD), y	64.23 (11.18)	62.88 (9.70)	65.94 (11.10)	63.89 (12.47)	.28
Female sex	128 (73.6)	10 (17.5)	44 (77.2)	44 (73.3)	.59
Educational level of high school or more	158 (90.8)	52 (91.2)	51 (89.5)	55 (91.7)	.89
Medical history					
Cardiac catheterization	41 (23.6)	12 (21.1)	14 (24.6)	15 (25.0)	.84
Claudication	16 (9.2)	6 (10.5)	6 (10.5)	4 (6.7)	.71
High blood pressure or hypertension	147 (84.5)	44 (77.2)	50 (87.7)	53 (88.3)	.18
High blood cholesterol level	100 (57.5)	32 (56.1)	35 (61.4)	33 (55.0)	.76
Congestive or chronic heart failure	12 (6.9)	6 (10.5)	4 (7.0)	2 (3.3)	.31
Stroke	6 (3.4)	3 (5.3)	2 (3.5)	1 (1.7)	.57
Ministroke or TIA	25 (14.4)	8 (14.0)	10 (17.5)	7 (11.7)	.66
Diabetes	62 (35.6)	21 (36.8)	18 (31.6)	23 (38.3)	.68
Chronic bronchitis or emphysema	20 (11.5)	7 (12.3)	5 (8.8)	8 (13.3)	.21
Asthma	27 (15.5)	9 (15.8)	5 (8.8)	13 (21.7)	.16
Cancer	22 (12.6)	9 (15.8)	5 (8.8)	8 (13.3)	.52
Kidney disease other than infection or a stone	9 (5.2)	5 (8.8)	2 (3.5)	2 (3.3)	.34
Stomach or duodenal ulcer	15 (8.6)	6 (10.5)	2 (3.5)	7 (11.7)	.24
Rheumatoid arthritis	25 (14.4)	7 (12.3)	7 (12.3)	11 (18.3)	.56
Arthritis other than rheumatoid	71 (40.8)	20 (35.1)	25 (43.9)	26 (43.3)	.57
No. of cigarettes smoked					
≥100 Cigarettes during lifetime	106 (60.92)	38 (66.67)	33 (57.89)	35 (58.33)	.57
1-4 Cigarettes/d	8 (13.11)	3 (11.11)	3 (15.00)	2 (14.29)	
5-15 Cigarettes/d	26 (42.62)	11 (40.74)	8 (40.00)	7 (50.00)	
1 Pack/d	17 (27.87)	8 (29.63)	7 (35.00)	2 (14.29)	
>1 Pack/d	6 (9.84)	2 (7.41)	2 (10.00)	2 (14.29)	
BMI, mean (SD)	32.93 (8.65)	31.61 (7.91)	32.75 (16.33)	34.37 (9.96)	.29
Systolic blood pressure, mean (SD), mm Hg	137.92 (17.24)	134.04 (17.05)	138.42 (18.16)	141.33 (16.04)	.049
ABI, mean (SD)	0.86 (0.14)	0.86 (0.14)	0.87 (0.14)	0.84 (0.15)	.47
Short physical performance battery score, mean (SD)^b^	7.67 (1.66)	7.74 (1.65)	7.82 (1.31)	7.47 (1.94)	.86
6-min Walking distance, mean (SD), m	357.55 (71.53)	366.72 (72.85)	350.15 (70.61)	355.85 (71.37)	.38
VascuQoL scores, mean (SD)^c^					
VascuQoL	5.40 (1.04)	5.57 (0.95)	5.21 (1.14)	5.42 (1.00)	.27
Activity	5.05 (1.01)	5.08 (0.97)	4.88 (1.11)	5.17 (0.94)	.38
Symptom	5.63 (1.11)	5.81 (1.02)	5.42 (1.19)	5.66 (1.11)	.17
Pain	4.70 (1.64)	5.11 (1.50)	4.49 (1.72)	4.51 (1.65)	.07
Emotional	5.89 (1.16)	6.09 (1.06)	5.69 (1.23)	5.90 (1.15)	.10
Social	6.08 (1.45)	6.10 (1.46)	5.89 (1.64)	6.23 (1.25)	.60
Treatment Self-regulation Questionnaire scores, mean (SD)^d^					
Intrinsic (autonomous)	6.32 (0.85)	3.28 (0.77)	6.34 (0.90)	6.35 (0.90)	.45
Extrinsic (external)	2.63 (1.42)	2.52 (1.36)	2.51 (1.42)	2.85 (1.48)	.36
Amotivation	2.36 (1.26)	2.51 (1.11)	2.24 (1.29)	2.34 (1.37)	.29
SF-12 score, mean (SD)^e^					
Physical health	41.87 (10.63)	42.77 (10.40)	40.22 (11.66)	42.59 (9.80)	.53
Mental health	51.61 (10.03)	52.12 (8.80)	50.79 (10.92)	51.92 (10.33)	.78

Abbreviations: ABI, ankle-brachial index; BMI, body mass index (calculated as weight in kilograms divided by height in meters squared); MI, motivational interviewing; PACE, Patient-Centered Assessment and Counselling for Exercise; SF-12, Medical Outcomes Study 12-Item Short Form Health Survey; TIA, transient ischemic attack; VascuQoL, Vascular Quality of Life.

^a^ Data are presented as number (percentage) of patients unless otherwise indicated.

^b^ Short physical performance battery score is measured on the 1- to 12-point scale in which a lower value indicates several limitations and a higher value indicates minimal limitations with physical performance.

^c^ The VascuQoL is scored on the scale from 1 to 7, with higher values indicating a better health status.

^d^ The Treatment Self-regulation Questionnaire is measured on a 1- to 7-point scale, with 1 indicating not at all true and 7 indicating very true.

^e^ The SF-12 is scored on a 0- to 100-point scale, with 0 indicating the most severe limitation and 100 indicating no limitation.

**Table 2.** Six-Month Change in Study Outcomes^a^

Outcome Measure	Mean (SE)		Change (95% CI)		*P* Value	6-mo With-MI Comparison Change (95% CI)	*P* Value
	Baseline	6-mo Follow-up	6-mo Within-Group Change	6-mo With-Control Comparison			
**6-min Walking Distance, m**							
MI	366.72 (9.65)	363.30 (9.88)	−3.42 (−12.38 to 5.54)	−2.10 (−16.54 to 12.35)	.78	1 [Reference]	NA
PACE	350.15 (9.35)	352.89 (10.31)	2.74 (−9.03 to 14.50)	2.31 (−11.36 to 15.97)	.74	4.41 (−9.94 to 18.75)	.55
Control	355.85 (9.21)	355.68 (9.15)	−0.18 (−8.83 to 8.48)	0 [Reference]	NA	NA	NA
**SF-12 Mental Health Score**^b^							
MI	52.11 (1.23)	53.82 (1.18)	1.71 (−0.19 to 3.61)	1.93 (−0.92 to 4.79)	.18	1 [Reference]	NA
PACE	50.45 (1.49)	53.24 (1.37)	2.79 (0.54 to 5.04)	2.37 (−0.62 to 5.37)	.12	0.44 (−2.67 to 3.56)	.78
Control	51.92 (1.33)	51.77 (1.46)	−0.15 (−2.26 to 1.97)	0 [Reference]	NA	NA	NA
**SF-12 Physical Health Score**^b^							
MI	42.37 (1.43)	41.74 (1.48)	−0.63 (−2.77 to 1.52)	1.07 (−4.84 to 4.50)	.54	1 [Reference]	NA
PACE	40.31 (1.55)	39.52 (1.73)	−0.79 (−3.82 to 2.25)	0.18 (−3.18 to 3.54)	.92	−0.89 (−4.51 to 2.74)	.63
Control	42.59 (1.27)	40.82 (1.45)	−1.77 (−3.89 to 0.35)	0 [Reference]	NA	NA	NA
**VascuQoL Score**^c^							
MI	5.57 (0.13)	5.82 (0.13)	0.25 (0.05 to 0.45)	0.25 (−0.02 to 0.53)	.07	1 [Reference]	NA
PACE	5.21 (0.15)	5.49 (0.14)	0.28 (0.07 to 0.49)	0.18 (−0.09 to 0.44)	.20	−0.08 (−0.36 to 0.20)	.58
Control	5.42 (0.13)	5.46 (0.14)	0.04 (−0.15 to 0.23)	0 [Reference]	NA	NA	NA
**Activity Score**^c^							
MI	5.08 (0.13)	5.45 (0.13)	0.37 (0.14 to 0.59)	0.35 (0.04 to 0.65)	.03^d^	1 [Reference]	NA
PACE	4.87 (0.15)	5.22 (0.15)	0.35 (0.08 to 0.63)	0.23 (−0.11 to 0.58)	.18	−0.11 (−0.48 to 0.26)	.55
Control	5.17 (0.12)	5.15 (0.13)	−0.02 (−0.22 to 0.19)	0 [Reference]	NA	NA	NA
**Emotion Score**^c^							
MI	6.09 (0.14)	6.21 (0.15)	0.13 (−0.13 to 0.39)	0.19 (−0.17 to 0.56)	.30	1 [Reference]	NA
PACE	5.68 (0.17)	5.94 (0.18)	0.26 (−0.07 to 0.60)	0.14 (−0.27 to 0.55)	.49	−0.05 (−0.48 to 0.38)	.82
Control	5.90 (0.15)	5.92 (0.16)	0.02 (−0.23 to 0.27)	0 [Reference]	NA	NA	NA
**Pain Score**^c^							
MI	5.11 (0.20)	5.53 (0.21)	0.41 (0.10 to 0.73)	0.36 (−0.07 to 0.80)	.10	1 [Reference]	NA
PACE	4.48 (0.23)	4.83 (0.23)	0.36 (0.03 to 0.68)	0.11 (−0.35 to 0.57)	.65	−0.26 (−0.73 to 0.22)	.29
Control	4.51 (0.21)	4.75 (0.22)	0.24 (−0.09 to 0.57)	0 [Reference]	NA	NA	NA
**Social Score**^c^							
MI	6.10 (0.19)	6.34 (0.15)	0.25 (−0.05 to 0.54)	0.28 (−0.15 to 0.71)	.20	1 [Reference]	NA
PACE	5.88 (0.22)	6.16 (0.18)	0.29 (−0.05 to 0.62)	0.18 (−0.24 to 0.60)	.40	−0.10 (−0.55 to 0.34)	.65
Control	6.23 (0.16)	6.11 (0.17)	−0.12 (−0.38 to 0.14)	0 [Reference]	NA	NA	NA
**Symptom Score**^c^							
MI	5.81 (0.13)	6.00 (0.14)	0.19 (−0.04 to 0.42)	0.25 (−0.04 to 0.55)	.09	1 [Reference]	NA
PACE	5.42 (0.16)	5.78 (0.16)	0.36 (0.12 to 0.59)	0.29 (−0.01 to 0.60)	.06	0.04 (−0.27 to 0.36)	.79
Control	5.66 (0.14)	5.64 (0.15)	−0.01 (−0.20 to 0.17)	0 [Reference]	NA	NA	NA

Abbreviations: MI, motivational interviewing; NA, not applicable; PACE, Patient-Centered Assessment and Counseling for Exercise; SF-12, Medical Outcomes Study 12-Item Short Form Health Survey; VascuQoL, Vascular Quality of Life.

^a^ Imputed data were used to replace the missing data.

^b^ The SF-12 is scored on a 0- to 100-point scale, with 0 indicating the most severe limitation and 100 indicating no limitation.

^c^ The VascuQoL is scored on the scale from 1 to 7, with a higher value indicating better health status.

^d^ Statistically significant (*P* < .05).

### Exploratory Analyses

In additional analyses (Table 3), walking distance increased at 6 and 12 months for participants older than 63.7 years who were randomized to PACE. In further exploratory analyses, participants randomized to PACE who completed at least 7 of the 9 counseling sessions had significantly increased walking distance at 6 and 12 months compared with control participants. Specifically, the mean (SE) change in walking distance at 6 months in the PACE group compared with the control group, with control for baseline walking distance, was 39.10 (15.46) m (*P* = .01). At 12 months, the mean (SE) increase in walking distance for the PACE participants who completed at least 7 counseling sessions was 48.28 (18.88) m (*P* = .01).

**Table 3.** Change in 6-Minute Walking Distance at 6 and 12 Months Among Subsets of Participants^a^

Characteristic	6-min Walking Distance Change, m, mean (95% CI)		
	MI (n = 57)	PACE (n = 57)	Control (n = 60)
**6-mo Follow-up**			
Median age, y			
≤63.7	1.51 (−14.01 to 10.99)	15.02 (−36.56 to 6.52)	3.43 (−9.62 to 16.48)
>63.7	6.05 (−18.20 to 6.10)	15.65 (4.06 to 27.24)	−3.55 (−14.91 to 7.81)
Median 6-min walking distance			
Less than or at baseline	1.91 (−13.10 to 16.93)	8.10 (−8.75 to 24.94)	5.19 (−6.20 to 16.58)
Greater than baseline	−6.78 (−17.11 to 3.56)	−2.44 (−19.94 to 15.05)	−7.69 (−20.69 to 5.32)
ABI			
<0.9	−8.97 (−22.26 to 4.31)	8.57 (−5.73 to 22.87)	−6.37 (−19.07 to 6.32)
≥0.9	0.61 (−11.13 to 12.35)	−1.21 (−18.61 to 16.19)	6.45 (−5.75 to 18.65)
Sex			
Female	−0.04 (−11.00 to 10.92)	5.58 (−8.12 to 19.29)	−0.44 (−10.73 to 9.84)
Male	−11.39 (−23.38 to 0.61)	−7.95 (−33.16 to 17.26)	0.56 (−33.16 to 19.13)
Diabetes			
Yes	−6.37 (−21.65 to 8.91)	5.61 (−9.35 to 20.58)	−8.37 (−19.04 to 2.31)
No	−2.88 (−13.74 to 7.99)	1.41 (−14.46 to 17.28)	5.42 (−7.14 to 17.97)
**12-mo Follow-up**			
Median age, y			
≤63.7	−6.43 (−21.50 to 8.64)	4.73 (−12.78 to 22.24)	7.06 (−11.64 to 25.75)
>63.7	9.56 (−25.06 to 5.95)	20.30 (4.77 to 35.84)	−8.69 (−22.25 to 4.87)
Median 6-min walking distance			
Less than or at baseline	0.84 (−19.02 to 20.71)	13.82 (−4.44 to 32.08)	4.30 (−10.12 to 18.72)
Greater than baseline	−13.15 (−25.58 to −0.71)	13.67 (−3.83 to 31.17)	−8.61 (−27.11 to 9.90)
ABI			
<0.9	−11.41 (−27.87 to 5.06)	17.08 (−4.17 to 38.32)	−14.67 (−29.29 to −0.06)
≥0.9	−5.08 (−19.47 to 9.30)	11.49 (−2.59 to 25.58)	13.46 (−3.96 to 30.87)
Sex			
Female	−2.84 (−15.24 to 9.57)	14.30 (1.59 to 27.02)	−2.78 (−16.73 to 11.17)
Male	−19.29 (−38.48 to −0.11)	11.65 (−18.77 to 42.08)	3.60 (−15.08 to 22.28)
Diabetes			
Yes	−3.91 (−23.46 to 15.65)	7.78 (−9.23 to 24.79)	−13.53 (−26.60 to −0.46)
No	−11.41 (−24.48 to 1.67)	16.50 (0.44 to 32.56)	6.55 (−9.77 to 22.87)

Abbreviations: ABI, ankle-brachial index; MI, motivational interviewing; PACE, Patient-Centered Assessment and Counseling for Exercise.

^a^ Imputed data were used to replace the missing data.

For participants completing the 6-month follow-up, those randomized to PACE had a mean (SD) increase in walking distance of 1.70 (46.81) m and those randomized to MI had an increase of −5.15 (29.93) m (*P* = .048). Similarly, for participants completing the 12-month follow-up, those randomized to PACE had a statistically significant mean (SD) increase in walking distance of 12.92 (41.00) m and those randomized to MI had an increase of −9.79 (40.14) m (*P* = .002). In addition, for participants completing the 12-month follow-up, PACE participants had a statistically significant increase in walking distance compared with control participants. Specifically, control participants had a mean (SD) change of −0.27 (44.48) m at 12 months (*P* = .04).

We analyzed the subgroup who met criteria for achieving a clinically meaningful improvement in walking distance of at least 20 m.^33^ We found a greater observed than expected frequency of improvement at 12 months in PACE vs MI participants.

### Adverse Events

Chest pain or shortness of breath that required hospitalization occurred among 5 participants (3 in the MI group, 1 in the PACE group, and 1 in the control group). Twenty-nine participants developed new orthopedic symptoms (9 in the MI group, 14 in the PACE group, and 6 in the control group). None of the hospitalizations were considered to be study related. All new orthopedic symptoms were considered to be study related.

## Discussion

Our results indicate that MI was not efficacious in improving walking distance in African American patients with PAD. Reasons for this are likely multifactorial, but some prior studies^34,35^ provide support for our findings. Miller and colleagues^34^ assessed perceptions of physical activity and MI (described as a more patient-centered counseling style) among rural African American women with type 2 diabetes and found that they had negative perceptions of MI’s patient centeredness. The women preferred the more traditional paternalistic approaches of counseling, which were also viewed as more familiar to the patients. Similarly, Levinson and colleagues^35^ assessed public preferences for participation in clinical decisions based on survey data from 2765 English-speaking respondents (mean [SD] age, 46.3 [17.4] years; 55.6% women, 14.5% African American, and 7.3% Latino). Women and persons with higher levels of education preferred a more active role in decision making. African American and Hispanic respondents preferred that physicians make decisions for them. Preferences for shared decision making increased with age until 45 years, when this preference decreased. Of note in our study, no statistically significant differences were found between groups in levels of autonomous or intrinsic motivation at baseline. However, the MI group had an intrinsic motivation score of 3.30 compared with 6.34 for the PACE group. Theoretically, MI should work by increasing intrinsic motivation, but its effect was negligible at 6 months and decreased at 12 months. Motivational interviewing did not have the anticipated effects on intrinsic motivation in the MI group despite this group’s relatively low levels of baseline motivation compared with the PACE group.

In contrast to MI, PACE showed promise for improving walking distance. PACE was more efficacious than MI at 12 months, with greater improvement in walking distance compared with control at 12 months. Among completers, PACE was efficacious for improving walking distance at 12 months compared with control or MI. Because PACE is a more scripted counseling approach and participants are largely told what to do, its potential efficacy aligns with the findings of Miller et al^34^ and Levinson et al^35^ noted above. PACE is a more practitioner-driven counseling style, allowing practitioners to spend most of the time talking, with participants largely listening. PACE’s prescriptive, solution-focused health education style may be more easily implemented in clinical settings where practitioners are accustomed to more expert-patient interaction.

Our work adds to the increasing body of literature on the efficacy of community-based walking therapy, also referred to as home-based exercise programs. These studies^36,37,38,39,40^ have had mixed results, with both positive and negative findings for home-based programs’ efficacy in improving walking distance. Participant demographics within the prior trials differed from our trial based on race and sex. Furthermore, for trials that demonstrated efficacy of home-based exercise programs, the interventions included more in-person contact time.

In regard to race and PAD, African American patients have a higher prevalence of PAD and are at higher risk for functional decline compared with non-Hispanic white patients.^7,18^ African American patients also have a higher rate of lower-extremity amputations.^6,41,42,43,44,45,46^ These findings exist even after controlling for multiple confounders, including access to care. Durazzo and colleagues^8^ published the results of a retrospective medical record review of a national hospital database of patients with a primary diagnosis of critical limb ischemia from 2002 to 2008 (N = 774 399). African American patients had greater odds of amputation compared with non-Hispanic white patients. Of interest, the racial disparity was greatest among hospitals with the greatest capacity to perform lower-extremity revascularization and among hospitals in wealthier zip codes. These findings highlight the need for interventions to address racial disparities in PAD outcomes. Early intervention may be one solution.

### Limitations

Our study has several limitations, including lack of a non-Hispanic white comparison group. In addition, our results may not be generalizable to participants who were ineligible and/or had more severe PAD. Another limitation is our lack of determining the anatomical level of PAD (eg, aortoiliac or femoropopliteal), which could influence a participant’s response to exercise therapy. Furthermore, we did not correct for multiple comparisons in our exploratory analyses. Although the finding was not statistically different, we had more dropouts in the PACE group at 6 months. Additional limitations are time and resource constraints imposed on physicians as part of their efforts to address behavior change during a clinic visit. Although our results did not demonstrate the efficacy of MI, the use of this counseling approach by primary care physicians could potentially add additional time to a visit, for which the length of such a visit may already be challenged by the needs of a patient with a complex condition. Furthermore, resources are needed to ensure that physicians are properly trained in the delivery of MI. Future studies that assess the efficacy of MI should also assess the added time to a given clinic visit and the cost of obtaining training to deliver MI.

## Conclusions

 In a cohort of African American patients with PAD, MI was not efficacious in improving walking distance at 6 or 12 months. The results of this study do not support the use of MI to improve walking performance in African American patients with PAD.

## Notes

Supplement 1. **Trial Protocol**

Supplement 2. **eTable.** Twelve-Month Change in Study Outcomes

Supplement 3. **Data Sharing Statement**
