# Supplementary material for: Efficacy of Community-Based Exercise Therapy Among African American Patients With Peripheral Artery Disease: A Randomized Clinical Trial
Source: JAMA Netw Open. 2019 Feb 15;2(2):e187959. doi: 10.1001/jamanetworkopen.2018.7959 (PMC6484888; doi:10.1001/jamanetworkopen.2018.7959)
Supplement: Supplement 1. — Trial Protocol [file jamanetwopen-2-e187959-s001.pdf]

Promoting Walking in African Americans with Peripheral Arterial Disease

Principal Investigator: Tracie Collins, MD

Protocol Version: 6.0, October 2012

PURPOSE

By doing this study, we will determine if motivational interviewing improves lower limb function in persons with poor leg circulation.

1. BACKGROUND

African Americans (AAs) are more than two times as likely as non-Hispanic whites to suffer from peripheral arterial disease (PAD) – atherosclerosis of the abdominal aorta and arteries of the lower extremities. Further, AAs with PAD suffer with greater walking impairment (defined as a reduction in walking distance, speed, and/or stair climbing) and more severe disease as compared to non-Hispanic whites. These identified disparities are largely attributed to lower levels of physical activity in AAs. Effective management of PAD among AAs is critically needed, particularly early in the course of the disease before the onset of severe morbidity (e.g., lower extremity amputation). Home-based walking is a potentially excellent therapy for PAD, but the patient must be motivated to walk. Because of low levels of physical activity, AAs with PAD have a particularly high need for motivational strategies to promote home-based walking.

Motivational interviewing is an effective counseling method in individuals who are less ready to change their behavior (i.e., low motivation). Thus, motivational interviewing is an ideal counseling method to promote home-based walking among AAs with PAD. In our pilot study to promote home-based walking, we used a counseling protocol, Patient-centered Assessment and Counseling for Exercise (PACE)[1] which targets known modifiable determinants of behavior change (e.g., social support). Using PACE, we demonstrated an improvement in stair climbing ability and lower limb blood flow (as measured by the ankle-brachial index). Improvements in these lower limb outcomes offer support for the use of PACE counseling in PAD, but PACE does not specifically address low motivation – a critical target for AAs with PAD. In addition, PACE is not culturally sensitive. In our proposed trial, we hypothesize that motivational interviewing will improve walking distance and reduce walking impairment more than PACE in AAs with PAD.

There is a critical need to improve lower limb outcomes in AAs with PAD. Our long-term goal is to reduce debilitating functional limitations and amputations in AAs with PAD. The overall objective of this application is to determine the most effective counseling strategy to improve home-based walking in AAs with PAD. We have robust pilot data to support the study hypotheses and ensure successful completion of the study.

We will deliver the PACE protocol or MI for 6 months, using both face-to-face visits and telephone contact. Our primary outcome is walking distance (as measured by the widely used and well-validated 6-minute walk test) at the end of active intervention (6 months). Secondary outcomes are walking distance as measured beyond the active phase of intervention (12 months), use of home-based walking (as measured by accelerometry),

and lower limb blood flow (as measured by the ankle brachial index - ABI). Our comparison group will receive the same print material as the two interventions as well as contact every three months to update any changes in contact information and to assess their health status. We will randomize 204 participants to one of three arms: Control (Tx1); PACE (Tx2); or MI (Tx3). In addition, we will determine the efficacy of PACE (Tx2) to increase walking distance in AAs with PAD, compared to Control (Tx1).

Primary Hypothesis:

1. At 6 months, AAs with PAD randomized to MI (Tx3) will have a greater increase in their walking distance, compared to those receiving PACE (Tx2) and the control group (Tx1).

Secondary Hypotheses:

1. AAs with PAD randomized to MI (Tx3) will have a greater increase in their walking distance at 12 months – a follow-up period beyond the six months of active intervention – compared to those receiving PACE (Tx2) and compared to the control group (Tx1).
2. At 6 and 12 months, AAs with PAD randomized to MI (Tx3) will have a greater increase in their home-based walking and their lower limb blood flow, compared to those receiving PACE (Tx2) and to the control group (Tx1).
3. At 6 and 12 months, AAs with PAD randomized to PACE (Tx2) will have a greater increase in their walking distance compared to those randomized to control (Tx1).

Exploratory Aim:

We will explore potential mediators (self-efficacy, social support, intrinsic/extrinsic motivation) and moderators (co-morbidities, leg symptom type, stage of change) of intervention effects on walking distance, home-based walking, and lower limb blood flow among AAs with PAD.

2. METHODS

Research Study Design:

We will conduct a 12-month, 3-arm randomized trial to determine the benefits of the 6-month long MI intervention –and culturally sensitive print material – in African Americans with PAD.

Pilot Study

The principal investigator was a faculty member at the University of Minnesota in Year 1 of the study. The pilot study was completed in Year 1. The pilot study served to assist the study team with recruitment strategies and to further develop the culturally sensitive print material. During the first year of the project, staff created and worked collaboratively with the Scientific Advisory Board and the Community Advisory Board to evaluate and advise regarding the components and delivery of the intervention and to prepare for the intervention clinical trial.

Clinical Trial

Year 2 – 5 will include the full trial, data analysis, and completion of initial manuscripts. Below, the study flow is outlined.

Step 1. Telephone Screening

For the initial step to assess eligibility, research staff will conduct a telephone screen of each potential participant. During the telephone interview, study staff will review the exclusion checklist and administer the Physical Activity Readiness Questionnaire (PAR-Q). The research staff will also gather contact information, demographics, and any co-morbidities (e.g. arthritis, COPD, asthma, angina, diabetes, and hypertension).

- The PAR-Q, developed by Canadian exercise experts, is a valid and proven tool for screening individuals prior to initiation of physical activity. The questionnaire detects relevant problems that may require further evaluation by a physician prior to starting any form of exercise.
- Patient-centered Assessment and Counseling for Exercise (PACE) assessment protocol will be used to identify persons who are in the Stage of Action (score in the range of 5 to 8). As noted in the exclusion criteria and given our focus on sedentary persons, we will exclude persons at such high levels of activity.

Step 2. In-Person Screening Examination

To determine if a participant has PAD, study staff will perform an ankle brachial index (ABI; a sensitive measure to diagnose PAD) and administer the Short Physical Performance Battery (SPPB) during the initial in-person visit. If the participant has PAD, he/she will complete a treadmill test. Each participant will also receive a culturally sensitive handbook for managing PAD and a pedometer.

The ABI may be done during mass screening events such as public health fairs or events at churches. Consent to join the study will not be obtained at these events. The test will be explained to anyone who approaches the booth and their verbal agreement to have the test done will be considered consent. If the results of the ABI test indicate the individual qualifies for the study, the study will be explained to them. If they are interested in joining the study, they will be asked to schedule an appointment to come in for the SPPB. Full study informed consent will be obtained at that visit.

There may also be situations where a potential participant contacts the study team with an interest in joining the study but cannot easily leave their home. In this event, the study team will go to the participant's home and perform the ABI test and the SPPB. A screening consent form has been developed for this situation. The study team will review this one page document with the potential participant and they will be asked to sign before any study activities are performed. If the participant passes this stage of screening and wishes to continue into the study, travel arrangements will be made for them to come in for the treadmill test.

- Ankle-Brachial Index (ABI): Eligibility and Secondary Outcome: A participant will rest for 5 minutes and a 5 or 8 MHz hand-held Doppler will be used to measure systolic blood pressures in both brachial arteries and in both ankles (i.e., the dorsalis pedis and posterior tibial arteries). The resting ABI will be calculated based on the ratio of the ankle and arm pressures. For each leg, the ankle pressure will be the higher of the dorsalis pedis and posterior tibial artery systolic blood pressures. The arm pressure will be the higher of the right and left brachial systolic pressures. The leg with the lowest ABI will be the determining cut-point

for defining disease. The ABI will be assessed at baseline and again at the 12-month follow-up visit. We will provide baseline training (10 hours) to all study staff, and refresher training every 6 months. Random fidelity checks of the staff will be performed by Dr. Collins.

- Short Physical Performance Battery (SPPB): This assessment is a powerful predictor of disability and mobility. The patient will be asked to do a series of timed-functional tasks. They will first be asked to attempt three balance tests, starting with their feet together (side by side) for 10 seconds. Then they will be asked to do semi-tandem stand (heel of one foot placed by the big toe of the other foot which they may put either foot in front, whichever is more comfortable for them) for 10 seconds. Final test is tandem stand (heel of one foot in front of and touching the toes of the other foot) for 10 seconds. If participant is unable to hold the position for each of the balance tests for 10 seconds, record result and move to gait speed test. For the gait speed test, the participant will be asked to walk 13 feet and one half inch at their usual speed, just as if they were walking down the street to go to the store. Lastly, the participant will be asked to fold their arms across their chest and sit, so that their feet are on the floor then stand up keeping their arms folded across their chest. If the participant is able to complete the initial chair stand, they will be asked to complete five continuous chair stands without using their arms. All of the functional tests are timed and scored based on the time. If the SPPB is not performed in the home as indicated above, the participants will complete this testing at KUMC-Wichita. Participants will be given parking directions and a map to indicate where this appointment will be held.
- Submaximal Exercise Treadmill Test: Clinical Safety to Engage in a Walking Program: PAD is a marker for atherosclerosis in other vascular beds, most notably the coronary arteries. Although many patients do not have significant coronary artery disease limiting daily activities, it is imperative to identify individuals who may experience exercise-induced coronary ischemic symptoms, or clinically silent exercise-induced ST depression > 2.0 mm, for whom study inclusion would be contraindicated. We will conduct a symptom-limited exercise test also known as a sub-maximal test. We will use an exercise treadmill test with 12-lead electrocardiographic monitoring and measurement of blood pressure. This graded exercise treadmill test requires a constant treadmill speed with modest increases in grade every few minutes. Specifically, the treadmill test will begin at 2 mph per participant's comfort level. The speed of the treadmill test may be reduced to no less than 1.5 mph. The incline increase will be a 2% increase of incline every 2 minutes. For participant's recovery, return to baseline vitals is the goal with a maximum time of 20 minutes. Following a demonstration by study staff, the patient will walk on a treadmill to maximal walking distance. During testing, patients will rate leg discomfort and rate of perceived exertion. Leg discomfort will be based on a scale of 1 to 4: 0=no pain, 1=onset of pain, 2=mild pain, 3=moderate pain, and 4=severe pain. Rate of perceived exertion will be based on the Borg scale. For the purposes of the study, the participant will be excluded if they cannot walk for a minimum of two minutes or if they have a Short Physical Performance Battery score of 11 or higher. If a participant is unable to complete two minutes on the treadmill on their first try, they will be asked to come back once to try the test again. This treadmill test will take place at a separate

visit from the ABI and SPPB. Participants will be asked to come to Heartland Cardiology for this testing which will be overseen by a Cardiologist and/or Exercise Physiologist. Again, participants will be given parking directions and a map to indicate where to go for this appointment.

Step 3: Enrollment/Randomization Visit

During the enrollment visit, scheduled within 2 weeks of the screening visit (Step 2), patients will complete the baseline 6-minute walk test, baseline blood draw, blood pressure, height, weight, complete all questionnaires (Barriers Self Efficacy, CHAMPS activities questionnaire for Older Adults, Fruit &Vegetable Intake, Fat Intake, Exercise Self-Efficacy, Lifestyle and Clinical Survey, San Diego Claudication Questionnaire, The SF-12 Health Survey, Social Support and Exercise Survey, Supplemental questions, Treatment Self-Regulation Questionnaire, VascQOL, and Walking Impairment Questionnaire), and undergo randomization.

Outcome Measures/Dependent Variables (Table 1):

- Primary Outcome Assessment: 6-Minute Walk Test
  The 6-minute walk test is the most widely accepted and objective measure of walking distance. In contrast to treadmill testing, it provides information on patients' ability to walk in the community, thus it is a useful measure of the functional outcomes of our behavioral intervention to promote home-based walking. The test is conducted by placing two cones 50 feet apart in a marked hallway and instructing patients to walk as many laps around the cones as possible. Patients are permitted to stop walking during the test, but recording of time will continue during the rest period. We will record time and distance to onset of leg discomfort, rate of perceived exertion at baseline, minute 2, minute 4 and post-test, total distance walked (feet). In a prior study involving 64 patients with PAD, the reliability coefficient for distance during 6-minute walk tests performed one week apart was 0.94 with a coefficient of variation of 11.7%[2].
- Secondary Outcome: Accelerometry-Measured Home-Based Walking:
  For patients who have met eligibility criteria, we will distribute an Actigraph accelerometer MODEL GT3XE (ActiGraph, Pensacola, FL) to objectively measure total physical activity and bouts of home-based walking. It uses an internal vertical plane accelerometer to measure both movement and intensity. The analog acceleration signal is processed by an analog-to-digital converter, producing a unit-less "count" value proportional to the number of movements (similar to the number of steps from a pedometer) and the velocity of those movements. The user specifies the time interval over which these values will be summed (10-second intervals for this study). Count values are stored in the ActiGraph's internal memory and uploaded to a computer for processing. The ActiGraph is lightweight, small, and worn on the waist, so it will detect whole-body movement and functions particularly well with walking activity. At the screening visit and at months 6 and 12, we will distribute accelerometers and a return envelope. Participants in all study arms will wear the ActiGraph monitor for 7 days at each of these three time points. Initially distributing the accelerometer at treadmill test session will allow for collection of accelerometer data prior to randomization.

By using a custom-developed program[3], ActiGraph data will be reduced to several summary variables excluding bouts of at least 60 minutes of continuous zeros indicative of times the monitor was not worn. At least three days of data will be needed to be included in the data set and all days with at least 10 hours of data. First, we will calculate average counts per day across all days. Second, we will classify ActiGraph counts into intensity categories of sedentary, light, and walking activity using individually determined ActiGraph count cutoffs based on count values obtained during the baseline 6-minute walk test. Because PAD patients have limited functional ability, we will not calculate a count cutoff distinguishing true moderate (3-5.9 times resting metabolic rate; 3-5.9 METs) or vigorous intensity physical activity (6 times resting metabolic rate; equivalent to a 5 mph jog). We will use individual-level cutoffs for this population since no cutoffs have yet been established for older adults with limited mobility. The individual cutoff approach has been supported for use in older adults and for intervention research. To determine sedentary and walking intensity count cutoffs, ActiGraphs will be set to record data in 10-second intervals. The cutoff between sedentary and light activity will be determined by having the subject sit quietly in a comfortable chair; the cutoff between light and walking intensity activity will be determined from the average count values obtained by asking the participant to walk for 3 minutes at their normal walking pace. The start and finish time will be recorded. We will use the shorter time interval (10 seconds vs. 1 minute) due to the intermittent nature of PAD patients' walking. Times when the patient is resting will not be used for calculation of count cutoffs.

- Quality of Life (QOL): The SF-12 and VascQOL questionnaires:
  The SF-12 is a shorter version of the SF-36 and has been validated in older AAs. It assesses general QOL. The VascQOL is disease specific and assess how each person has been affected by the poor circulation in their legs over the past two weeks.
- Supplemental Questions:
  There are six additional questions that ask about decision making behavior, unfairness, and the participant's insight on his/her standing in the community (i.e., self-perception).

Table 1. Measures

Variables	Measure	Description and Data Source	Data Collection Timepoints			Depend-ent Variable	Potential Moderator	Potential Mediator
**Outcomes**			Baseline	6 Months	12 Months			
Walking Distance	Six minute walking test	Objective measure of walking distance	X	X	X	X		
Home-Based Walking	Accelerometry	Objective measure of walking adherence	X	X	X	X		
Lower Limb Blood Flow	Ankle Brachial Index*	Objective measure of lower limb blood flow	X		X	X		
**Patient Variables**								
Sociodemographics,Co-existing illnesses, Social habits	Lifestyle and Clinical Survey	Survey	X				X	
Physical Activity	Community health Activities Model Program for Seniors (CHAMPS) Questionnaire	Survey	X	X	X		X	
Stage of Change	PACE Score	Survey	X	X	X		X	
Leg Symptoms	San Diego Claudication Questionnaire	Leg Symptom Categories; Survey	X	X	X		X	
Risk Factor Control	Laboratory data; blood pressure measurements; self-report of smoking cessation	Blood draws; Survey	X	X	X		X	
**Psychosocial Factors**		Survey						
Self-efficacy	Barriers Self Efficacy and Exercise Self Efficacy	Surveys	X	X	X			X
Social Support	Social Support Questionnaires : Friend Support for Exercise Scale Family Support for Exercise Scale	Surveys	X	X	X			X
**Cognitive Factors**								
Intrinsic Motivation	Treatment Self-Regulation Questionnaire (TSRQ).	Survey	X	X	X			X

* Following recommendation from the Scientific Advisory Board in year 1, the ankle- brachial index will only measure at baseline and 12 months

Potential Mediators

- Self-Efficacy for Exercise: Barriers Self-Efficacy and Exercise Self-Efficacy: We will utilize two measures to assess barriers to self-efficacy. The Barriers Self-Efficacy Scale, a 13-item measure, was designed to assess participants' perceived capabilities to exercise three times per week over the next 2 months while facing common barriers. Participants indicate their degree of confidence for

each item on a 0-100% scale. Internal consistency of this measure is high (α =0.92). The Exercise Self-Efficacy measure, composed of 8 items, captures a participant's efficacy for continued exercise participation (i.e., at least three times per week for 40 minutes) over incremental week periods for 8 weeks. The internal consistency of this measure is also high (α =0.92). The confidence scores from the above measures will be summed and divided by the total number of items giving a possible range of 0-100%. These two measures will be combined to provide a summary score of overall exercise efficacy.

- Social Support:
  The Social Support for Exercise Scale[4] contains 13 items describing a supportive behavior and assessing the extent to which friends and family demonstrate this support using a 5-point Likert scale (ranging from 1=none to 5=very often). This scale then is used to derive two subscales, one describing family support and one describing support from friends. This scale has been shown to demonstrate sufficient construct validity and reliability[4]. Criterion-related validity has also been reported in that social support for PA has been significantly associated with actual PA (r =.23 to r =.46)[4].
- Intrinsic-Extrinsic Motivation:
  Intrinsic motivation is a key concept in our theoretical model and it will be assessed with the Treatment Self-Regulation Questionnaire (TSRQ). The 15-item measure yields two main subscales of reasons why a respondent might either begin or maintain exercise: a) Intrinsic (Autonomous) Motivation, and b) Extrinsic (Controlled) Motivation.

Potential Moderators

- Co-morbidities/Symptomatology:
  - We will use the Lifestyle and Clinical Survey (LCS) to obtain sociodemographic (e.g., age) and comorbidity data. Dr. Collins originally developed the LCS to obtain pertinent past medical history and sociodemographic information, which could then be adjusted for in risk factor assessments for PAD within a cohort study. The survey was interviewer-administered to 30 patients and required no longer than 15 minutes to complete. Reliability evidence was excellent, with a summary kappa statistic of 0.81 (95% CI 0.78, 0.84). Validity evidence was very good, with a summary kappa statistic of 0.58 (95% CI 0.52, 0.64).
  - The San Diego Claudication Questionnaire (SDCQ), an expansion of the original World Health Organization/Rose Questionnaire, is a 9-item questionnaire for categorizing leg symptoms (no pain, atypical leg pain, intermittent claudication). The SDCQ asks about pain in either calf, thigh, or buttock on walking, whether the pain is present at rest, whether it ever disappears during walking, and whether the pain is relieved within 10 minutes or less upon stopping. It takes approximately 5 minutes to complete. Leg symptom subtypes will be analyzed as potential moderators.
  - We will determine atherosclerotic risk factor control. For smoking habits, we will ascertain current and past smoking habits. For glucose and lipid control, we will complete blood draws for glycosylated hemoglobin and

lipid profiles (fasting). For blood pressure control, we will obtain three serial blood pressure measurements, each separated by 2 minutes. The results will be averaged.

  - We will use the Community Healthy Activities Model Program for Seniors (CHAMPS) Questionnaire to assess physical activity. We will administer the 42-item questionnaire, designed for use among older persons, at baseline, 6, and 12 months to all three groups to assess frequency and duration of various physical activities typically undertaken by adults. Among a sample with a mean age of 74 years, 6-month stability of this instrument ranged from 0.58- 0.67, using intraclass correlation coefficients. All measures were sensitive to change with a $P < 0.01$. This questionnaire was validated for use in African Americans.

- Fruit & Vegetable Intake and Fat Intake:
  These two intakes will be used to assess dietary habits. The Fruit & Vegetable Intake asks participants to recall their servings of specific fruits and vegetables over the last week. The Fat Intake asks participants to recall specific foods that they have eaten over the past month. Both intakes will be assessed at baseline, 6-months, and 12-months.
- Walking Impairment Questionnaire (WIQ):
  The WIQ is a disease-specific questionnaire validated in patients with PAD. It consists of four subcategories: pain, distance, walking speed, and stair climbing. WIQ will be assessed at baseline, 6-months, and 12-months.
- Stage of Change:
  The Patient-Centered Assessment and Counseling for Exercise (PACE) score will be used to identify a participant’s stage of readiness for exercise. To obtain a PACE score, a participant chooses one of eight graded statements that best describe his/her current level of and interest in physical activity. This score determines the “Stage of Change” that they are in[5].

PACE and MI interventions:
Both interventions will consist of nine sessions that occur between randomization and the 6-month follow-up. There will be four in-person and five telephone sessions with a trained counselor. The MI and PACE sessions will be audio-taped and they may also be videotaped for fidelity purposes.

Patient-Centered Assessment and Counseling for Exercise (PACE):
PACE is a program based on the "stages of change" model and is designed to increase social support and self-efficacy, reduce perceived barriers to activity, and increase awareness of the benefits of activity. PACE requires participants to complete an assessment of their willingness to change. Based on their score, the participant receives a stage-matched protocol (Getting Out of Your Chair, Planning the First Step, or Keeping the PACE) to partially complete before seeing the counselor. During the counseling session, participant and counselor review the protocol and discuss stage-relevant information. The goals and discussion are follow-up up on at the next counseling session.

Motivational Interviewing (MI):

MI is a directive, client-centered counseling approach to elicit behavior change by assisting clients in exploring and resolving ambivalence. MI is best suited for persons who exhibit lower intrinsic motivation and readiness for behavior change. Within the philosophy behind MI, client resistance is often a behavior evoked by environmental conditions and is not necessarily indicative of intrinsic motivation to change. Thus, the counselor will engage the client in exploring resistance or ambivalence rather than combat it. The five main components of MI are: evocation, collaboration, autonomy, direction, and empathy. The counselors proactively try to evoke the client's own reasons for change and ideas about how change should happen. The counselor will also use collaboration as part of the MI sessions by encouraging power sharing in their interaction where the client's ideas will set the tone of the session. Another key component of MI is autonomy. Counselors are trained to elicit comments from the client that lead to a greater perceived choice regarding the target behavior (walking). MI is not only used for physical activity, but any behavior change with a targeted direction. It is important for the MI counselors to direct the client toward the target behavior. The counselor keeps the sessions on topic and does not allow the client to wander from the target behavior. The final key component of MI is empathy. The counselor will demonstrate a genuine understanding for what the client says and where they are coming from. The ultimate goal is to explore the individual's feelings about his or her walking without pressuring for behavior change. As previously mentioned, the MI sessions will be audio-taped. Random sessions will be coded by a trained MI instructor, in order to ensure validity among all MI counselors.

Control Arm:
In addition to the baseline, 6-month, and 12-month assessments, the control arm will only receive phone calls at months three and nine. The purpose of these phone calls is to get a general health update and to thank them for their participation in the study.

Follow-up:
We will assess all patients during in-person visits at 6- and 12 months. During the follow-up in-person visits, participants will be asked to complete the 6-minute walk test, a blood draw (lipid panel, A1C, and 60 ccs for stored blood), blood pressure, height, weight, and all questionnaires except the Lifestyle and Clinical Survey (LCS), which is a medical history questionnaire intended for use only at baseline. We will also capture accelerometer data during each follow-up visit. At 6- and 12 months, we will give patients the accelerometer and an envelope to mail it back to our study team. The morning after the 6- and 12-month follow-up assessments, we will call the patient with a reminder to wear the device. They will be called again on days 3 and 7 to answer questions. ABI will be re-assessed at the 12 month follow-up.

Inclusion Criteria
1. African American (determined by self-report)
2. Lived most of their life in the United States
3. Resting ABI < 0.995 – to assess for PAD
4. English speaking
5. Has a telephone – required for delivery of the intervention

Exclusion Criteria

1. Currently walking for exercise at least 5 days per week (i.e., a PACE score ranging from 5-8); the rationale is that a person who is currently walking for at least 5 days per week is already sufficiently active and therefore not a member of the target population for our motivational home-based walking intervention.
2. Prior major amputation (foot or lower leg) or critical leg ischemia (tissue loss, gangrene, or ulcers)
3. Rest pain with ABI <0.4 and non-palpable femoral pulses without prior evaluation by a vascular surgeon, given the need for evaluation for the role of more invasive therapy prior to recommending walking therapy
4. Leg revascularization within 3 months of enrollment or plans for revascularization during the study period; the rationale is that post intervention recovery and potential complications are likely to limit the patient's ability to adhere to the study protocol.
5. Use of supplemental oxygen; the rationale for this is concern for participant safety and potential limited ability to participate in the study secondary to breathing difficulty.
6. Myocardial infarction within the preceding 3 months; the rationale for this is participant safety and the potential risk for complications and/or the need for supervised cardiac rehabilitation following the event.
7. Resting blood pressure > 200/110 mmHg; the rationale for this is participant safety, as blood pressure may further increase during exercise and increase risk for a cerebrovascular event or myocardial infarction.
8. Exercise-induced coronary ischemic symptoms, or exercise-induced ST depression > 2.0 mm; the rationale for this is participant safety and the need for further cardiac evaluation prior to involvement in walking therapy[6].
9. Inability to walk for 2 minutes; the rationale being that people who cannot walk for 2 minutes would not be able to complete the necessary submaximal treadmill test, which is used to screen for coronary ischemic symptoms. We will also exclude anyone who can walk for 20 minutes or more during the submaximal treadmill test. Anyone who can complete the submaximal test would not have significant walking impairment and would not get that much out of the study. Short Physical Performance Battery score of 11 or higher as such persons do not have a clinically significant impairment in mobility; therefore, we will exclude anyone who scores a 11 or higher (out of a maximum of 12 points).

Recruitment Strategies

We will post flyers and brochures at local sites, including clinics, senior centers and churches within Wichita. We will obtain permission from appropriate leaders prior to posting or displaying any material. Letters will be sent to local physicians and church and community center leaders notifying them of the study and asking for referrals. A mass mailing postcard will also be sent out. In addition, we will use local newspaper and radio advertisements. The study will also be advertised on the KUSM-W facebook page, through a broadcast email that goes to KU faculty, students and staff, and in the Jayhawk Talk Online publication. All advertisements have been developed with the KU Public Affairs Office and will be submitted for IRB review.

The study team will attend local and regional health fairs and other community events in an effort to recruit participants. The ABI screening will be performed at these events.

Retention Strategies

A variety of items will be given or mailed to participants to keep them informed and engaged in the study. Participants and their physicians will receive letters updating them on the lab results and treadmill results that are obtained as part of study procedures. Participants will also receive Thank you and Birthday postcards throughout the study as applicable. In addition, participants will be given study brochures and handouts with PAD and study specific information and a card with important phone numbers.

Informed Consent Process

Participants will call in response to advertisements or physician referrals. Verbal consent will be obtained from study staff for the phone screening and for the ABI done as part of mass screening events. Written consent will be obtained by study staff when the participants come to the initial in-person screening. If the participant is seen in their home for the ABI and SPPB, they will first sign the screening consent form and if they qualify for the study, they will sign the full study consent before completing the treadmill test. Participants will have an opportunity to review the consent form and ask questions prior to signing. Participants will not be coerced into signing but they will be notified that participation in the study cannot proceed without a signed consent form.

Sample Size Justification

Sample size and power calculations used the common sample size formula for normally distributed statistics with a type I error level of α (2-sided test) and type II error of β:

$$n = \frac{V*(z_{1-\alpha/2} + z_{1-\beta})^2}{\Delta^2}$$

where Δ denotes the minimal meaningful difference to detect and V denotes the variance of the test statistic. For the ANCOVA analyses, V=2*σ2(1-ρ2) where σ2 is the (average) variability of the 6-minute walking distance at baseline and follow-up and ρ is the (average) correlation between baseline and follow-up measurements.136 The estimates for the sample size required in each arm are presented in Table 2 across a range of possible values for the correlation. These are based on a clinically meaningful difference to detect of one half of a city block (40 meters) and using a Bonferroni correction for the multiple comparisons, i.e., α was taken as 0.025. An estimate for the standard deviation was taken as approximately 88, which is based on the variability observed in a study reporting changes over 6 months in a similar population with PAD (Gardner 2001).[24]

Table 2. Sample size in each arm for desired power with corresponding correlation between measurements

	Correlation					
	0.60	0.65	0.70	0.75	0.80	0.85
80% Power	59.1	53.4	47.1	40.4	33.3	25.6

90% Power	77.2	69.7	61.5	52.8	43.4	33.5
97.5% Power	109.8	99.1	87.5	75.1	61.8	47.6

Based on a correlation of 0.75, we will have 92% power for each of the primary hypotheses with 57 patients in each arm having complete follow-up. If the correlation is as low as 0.65, we will have 83% power. While the calculations above assume homoscedasticity, this is for sample size and power estimation purposes. Analyses will use robust variance estimation for confidence intervals and P-values. Supportive analyses adjusting for potential imbalances in baseline variables between treatment groups will also be considered for added precision. General feasibility for adaptation to standard clinical practice will be evaluated by considering both an estimate of effectiveness, taking into account the level of counseling interaction received, as well as an estimate of efficacy for the planned frequency and duration of patient interaction. Attrition will be a potential limitation to the interpretation and generalizability of results. The planned enrollment of 204 is inflated to balance potential attrition. We expect to be able to hold attrition to no more than 15%. However, if we observe a rate as high as 20% we will still have 91% power with a correlation of 0.75 and 80% power with a less optimistic correlation of 0.65. Thus, in the event that we observe both a lower correlation and 33% higher attrition than expected, this study will still have 80% power to detect the outcome of interest. Extensive efforts to minimize the amount of missing data are summarized in section D5. Despite our best efforts, some missing data is likely to be unavoidable. If the missingness is missing completely at random, the consequence will merely be lost precision. If the data are missing at random, conditioned on measured covariates, then supplementary analyses adjusting for these covariates will produce unbiased results. For missing data mechanisms beyond measured covariates, we will examine the extent to which results may be affected. Multiple imputation will also be considered for missing data issues.

3. BENEFITS AND RISKS OF RESEARCH

Known and Anticipated Risk

We will use counseling strategies (i.e., stage of readiness to change approach or motivational interviewing techniques) and survey data for our interventions and data collection, respectively. Our counseling strategies have been used in prior studies without harm to participants but there could be unforeseen anxiety that arises from receipt of these strategies or with completing survey questions. We will be very cognizant of participant perceptions of counseling techniques and/or survey questions in an effort to attenuate any anxiety related to our study protocols.

Potential additional risks to participants:

- Unforeseen anxiety from discovering they have atherosclerosis in one or more vessels of their body.
- Increased fatigue from the use of routine exercise. This fatigue will subside as they become more accustomed to exercising.
- Increased leg pain from the use of routine exercise.

- Having blood drawn may lead to soreness at the site or the development of a bruise. The risk from this blood draw is the same as the risk as when blood is removed for routine laboratory work.

Anticipated Benefit

The potential benefits to subjects in either Tx2 or Tx3 are an increase on physical activity and reduction of walking impairment from PAD. There are no expected benefits for the control group.

4. MONITORING AND DISCONTINUATION

Plan for Monitoring and Reporting Unanticipated Problems

We designed the screening criteria with great care and consideration in order to exclude those participants who could not safely engage in a walking study. Throughout the study we will actively screen for adverse events every three months. We will also have an adverse event hotline that participants can call at any time. The statistician will regularly pull reports to look for patterns of adverse events. The PI will review events that require more immediate attention to determine the appropriate care and reporting. A Data Safety Monitoring Board (DSMB) will meet every six months. However, the frequency may vary depending on participant enrollment and frequency and severity of adverse events. Any adverse events will be reported to the local IRB and the DSMB simultaneously. A follow-up report will be submitted to the IRB to further clarify if the event has been determined related to the study by the DSMB.

Study Withdrawal/Discontinuation Procedures

During the consenting process, participants will be informed that, at any time, they can withdraw from the study.

5. PLAN FOR ASSURING PARTICIPANTS' PRIVACY AND CONFIDENTIALITY, FOLLOW-UP and RECORD RETENTION ISSUES

Confidentiality

The data will be entered and stored on a password-protected computer at KUSM-W, secured under lock and key with access restricted to research personnel only. In addition, HSC2 and government and regulatory bodies will have access, as required by law.

Privacy

The consent interview will be conducted in private to protect the conservation from being heard by others.

All study participants will be randomly assigned a study number. This number will not be associated with any identifying participant characteristics such as date of birth, social security number or medical record number. Any identifying participant information and

their assigned study number will be kept as a separate list that will be maintained in a secure location. No data forms will contain any specific participant identifiers. All data collected will be evaluated and analyzed only as group data and no specific participants will be identified in presentation or publication of study results. Once the study is completed and all manuscripts have been accepted, the master list will be destroyed and all computer files of the participant master list will be deleted.

References

1. Patrick K, Sallis J, Long B, Calfas K, Wooten W, Heath G. A new tool for encouraging activity: Project PACE. 22 ed. 1994. p. 45-52.

2. Montgomery PS, Gardner AW. The clinical utility of a six-minute walk test in peripheral arterial occlusive disease patients. 46 ed. 1998. p. 706-11

3. Sirard JR, Riner WF, Jr., McIver KL, Pate RR. Physical activity and active commuting to elementary school. Med Sci Sports Exerc 2005 Dec;37(12):2062-9.

4. Sallis JF, Grossman RM, Pinski RB, Patterson TL, Nader PR. The development of scales to measure social support for diet and exercise behaviors. Prev Med 1987 Nov;16(6):825-36.

5. Prochaska JO, DiClemente CC. Stages and processes of self-change of smoking: toward an integrative model of change. J Consult Clin Psychol 1983 Jun;51(3):390-5.

6. Hirsch AT, Haskal ZJ, Hertzer NR, Bakal CW, Creager MA, Halperin JL, et al. ACC/AHA 2005 Practice Guidelines for the management of patients with peripheral arterial disease (lower extremity, renal, mesenteric, and abdominal aortic): a collaborative report from the American Association for Vascular Surgery/Society for Vascular Surgery, Society for Cardiovascular Angiography and Interventions, Society for Vascular Medicine and Biology, Society of Interventional Radiology, and the ACC/AHA Task Force on Practice Guidelines (Writing Committee to Develop Guidelines for the Management of Patients With Peripheral Arterial Disease): endorsed by the American Association of Cardiovascular and Pulmonary Rehabilitation; National Heart, Lung, and Blood Institute; Society for Vascular Nursing; TransAtlantic Inter-Society Consensus; and Vascular Disease Foundation. Circulation 2006 Mar 21;113(11):e463-e654.
