# Supplementary material for: Efficacy of Community-Based Exercise Therapy Among African American Patients With Peripheral Artery Disease: A Randomized Clinical Trial
Source: JAMA Netw Open. 2019 Feb 15;2(2):e187959. doi: 10.1001/jamanetworkopen.2018.7959 (PMC6484888; doi:10.1001/jamanetworkopen.2018.7959)
Supplement: Supplement 2. — eTable. Twelve-Month Change in Study Outcomes [file jamanetwopen-2-e187959-s002.pdf]

Supplementary Online Content

Collins TC, Lu L, Ahluwalia JS, et al. Efficacy of community-based exercise therapy among African American patients with peripheral artery disease: a randomized clinical trial. *JAMA Netw Open*. 2019;2(2):e187959. doi:10.1001/jamanetworkopen.2018.7959

eTable. Twelve-Month Change in Study Outcomes

This supplementary material has been provided by the authors to give readers additional information about their work.

© 2019 Collins TC et al. *JAMA Network Open.*

eTable. Twelve-Month Change in Study Outcomes

Outcome Measures	Mean (SE)			Change (95% CI)				
	Baseline	12-Month Follow-up		12-Month Within-Group Change	12-Month With-Control Comparison	p-value	12-Month With-MI Comparison	p-value
**6-min Walking Distance [a]**								
MI	366.72(9.65)	358.98(9.82)		-7.75(-18.54 to 3.05)	-5.56 (-21.18 to 10.06)	0.49	0 (Reference)	NA
PACE	350.15(9.35)	363.90(10.99)		13.75(1.69 to 25.80)	14.24 (-1.85 to 30.34)	0.08	19.80 (3.33 to 36.28)	0.02*
Control	355.85(9.21)	354.78(9.86)		-1.08(-12.31 to 10.16)	0 (Reference)	NA	NA	NA
**SF-12 [b] Mental health T-score**								
MI	52.11 (1.23)	51.78 (1.31)		-0.33(-2.74 to 2.08)	-0.63(-3.73 to 2.47)	0.69	0 (Reference)	NA
PACE	50.45 (1.49)	53.55 (1.14)		3.1(1 to 5.20)	1.9(-1.01 to 4.81)	0.20	2.53(-0.66 to 5.73)	0.12
Control	51.92 (1.33)	52.32 (1.26)		0.4(-1.59 to 2.39)	0 (Reference)	NA	NA	NA
**SF-12 [b] Physical health T-score**								
MI	42.37 (1.43)	43.48 (1.53)		1.11(-1.36 to 3.59)	0.39(-3.29 to 4.07)	0.83	0 (Reference)	NA
PACE	40.31 (1.55)	38.51 (1.6)		-1.8(-4.36 to 0.76)	-3.34(-6.93 to 0.24)	0.07	-3.73 (-7.34 to -0.12)	0.04*
Control	42.59 (1.27)	43.22 (1.53)		0.63(-1.97 to 3.24)	0 (Reference)	NA	NA	NA
**VascuQoL Score [c]**								
MI	5.57 (0.13)	5.94 (0.11)		0.38(0.19 to 0.57)	0.13(-0.13 to 0.4)	0.33	0 (Reference)	NA
PACE	5.21 (0.15)	5.53 (0.13)		0.32(0.13 to 0.50)	-0.08(-0.36 to 0.2)	0.59	-0.21(-0.49 to 0.07)	0.14
Control	5.42 (0.13)	5.73 (0.13)		0.31(0.12 to 0.50)	0 (Reference)	NA	NA	NA
**Activity Score [c]**								
MI	5.08 (0.13)	5.48 (0.12)		0.39(0.16 to 0.62)	0.11(-0.22 to 0.45)	0.50	0 (Reference)	NA
PACE	4.87 (0.15)	5.26 (0.17)		0.40(0.11 to 0.70)	0(-0.39 to 0.39)	1.00	-0.11(-0.5 to 0.27)	0.56
Control	5.17 (0.12)	5.4 (0.14)		0.24 (-0.02 to 0.49)	0 (Reference)	NA	NA	NA
**Emotion Score [c]**								
MI	6.09 (0.14)	6.46 (0.14)		0.37 (0.11to 0.63)	0.32(-0.09 to 0.72)	0.12	0 (Reference)	NA
PACE	5.68 (0.17)	6.01 (0.19)		0.33 (0.01 to 0.65)	0.07 (-0.36 to 0.51)	0.74	-0.24(-0.66 to 0.17)	0.24
Control	5.9 (0.15)	6.04 (0.18)		0.15 (-0.15 to 0.45)	0 (Reference)	NA	NA	NA
**Pain Score [c]**								

© 2019 Collins TC et al. *JAMA Network Open.*

MI	5.11 (0.2)	5.47 (0.19)		0.36(0.01to 0.70)	-0.12(-0.61 to 0.37)	0.64	0 (Reference)	NA
PACE	4.48 (0.23)	4.74 (0.23)		0.26(-0.07 to 0.59)	-0.48 (-0.93 to -0.02)	0.04*	-0.36(-0.84 to 0.12)	0.14
Control	4.51 (0.21)	5.23 (0.22)		0.72(0.39 to 1.05)	0 (Reference)	NA	NA	NA
**Social Score** [c]								
MI	6.1 (0.19)	6.56 (0.12)		0.46(0.22 to 0.71)	0.3(-0.11 to 0.71)	0.15	0 (Reference)	NA
PACE	5.88 (0.22)	6.06 (0.19)		0.19(-0.16 to 0.53)	-0.14(-0.59 to 0.31)	0.54	-0.44(-0.9 to 0.02)	0.06
Control	6.23 (0.16)	6.29 (0.17)		0.06(-0.24 to 0.37)	0 (Reference)	NA	NA	NA
**Symptoms Score** [c]								
MI	5.81 (0.13)	6.08 (0.15)		0.27(0 to 0.53)	0.16(-0.2 to 0.52)	0.39	0 (Reference)	NA
PACE	5.42 (0.16)	5.65 (0.16)		0.23(-0.04 to 0.5)	-0.04(-0.41 to 0.33)	0.83	-0.2(-0.57 to 0.17)	0.29
Control	5.66 (0.14)	5.83 (0.16)		0.17(-0.07 to 0.42)	0 (Reference)	NA	NA	NA

*Significant (p-value < 0.05)
Imputed data were used to replace the missing data.
Abbreviations: SF-12, Medical Outcomes Study Short-Form Health Survey; VascuQoL, Vascular Quality of Life Scores.
[a] 6-min walking distance was measured in units of meters.
[b] SF-12 is used to measure health-related quality of life. The SF-12 is scored on a 0- to 100-point scale in which 0 indicates the most severe limitation and 100 indicates no limitation.
[c] VascuQoL is scored on the scale from 1 to 7. A higher value indicates better health status.

© 2019 Collins TC et al. *JAMA Network Open.*
