# Supplementary material for: Efficacy of Community-Based Exercise Therapy Among African American Patients With Peripheral Artery Disease: A Randomized Clinical Trial
Source: JAMA Netw Open. 2019 Feb 15;2(2):e187959. doi: 10.1001/jamanetworkopen.2018.7959 (PMC6484888; doi:10.1001/jamanetworkopen.2018.7959)
Supplement: Supplement 3. — Data Sharing Statement [file jamanetwopen-2-e187959-s003.pdf]

Data Sharing Statement

Collins. Efficacy of Community-Based Exercise Therapy Among African American Patients With Peripheral Artery Disease. *JAMA Netw Open*. Published February 15, 2019. 10.1001/jamanetworkopen.2018.7959

Data
Data available: Yes
Data types: Deidentified participant data
How to access data: Data request must be sent to tcollins2@kumc.edu
When available: With publication

Supporting Documents
Document types: None

Additional Information
Who can access the data: Data will be made available to researchers whose proposed use of the data has been approved.
Types of analyses: The data will be made available for research purposes that are clearly articulated.
Mechanisms of data availability: Data will be made available after approval of a proposal and with a signed data access agreement.
